# Mindfulness Interventions Delivered by Technology Without Facilitator Involvement: What Research Exists and What Are the Clinical Outcomes?

**DOI:** 10.1007/s12671-016-0548-2

**Published:** 2016-06-02

**Authors:** James Fish, James Brimson, Siobhan Lynch

**Affiliations:** 1 Academic Centre, Faculty of Life Sciences and Medicine, 1st Floor, Henriette Raphael Building, Guy’s Campus, London, SE1 1UL UK; 2 Institute of Psychiatry, Psychology and Neuroscience, King’s College London, 16 De Crespigny Park, London, SE5 8AF UK; 3 Medical Education, Faculty of Medicine, University of Southampton, England, UK

**Keywords:** Mindfulness, Meditation, Technology, Web-based, Systematic review, Facilitator, Clinical, Technological platform, Online, MBSR, MBCT

## Abstract

New cost-effective psychological interventions are needed to contribute to treatment options for psychiatric and physical health conditions. This systematic review aims to investigate the current literature on one potentially cost-effective form of mindfulness-based therapy, those delivered through technological platforms without any mindfulness facilitator input beyond the initial design of the programme. Three electronic databases (Ovid Medline, PsychINFO and Embase) were searched for relevant keywords, titles, medical subject headings (MeSH) and abstracts using search terms derived from a combination of two subjects: ‘mindfulness’ and ‘technology’. Overall, ten studies were identified. The majority of studies were web-based and similar in structure and content to face-to-face mindfulness-based stress reduction courses. Clinical outcomes of stress (*n* = 5), depression (*n* = 6) and anxiety (n = 4) were reported along with mindfulness (*n* = 4), the supposed mediator of effects. All eight studies that measured significance found at least some significant effects (*p* < .05). The highest reported effect sizes were large (stress *d* = 1.57, depression *d* = .95, both *p*s > .005). However, methodological issues (e.g. selection bias, lack of control group and follow-up) which reflect the early nature of the work mean these largest effects are likely to be representative of maximal rather than average effects. Whilst there are important differences in the construction, length and delivery of interventions, it is difficult to draw firm conclusions about the most effective models. Suggestions of key characteristics are made though, needing further investigation preferably in standardised interventions. Given the existing research and the speed at which technology is making new platforms and tools available, it seems important that further research explores two parallel lines: first, refinement and thorough evaluation of already established technology-based mindfulness programmes and second, exploration of novel approaches to mindfulness training that combine the latest technological advances with the knowledge and skills of experienced meditation teachers.

## Introduction

There is now a great deal of evidence supporting mindfulness-based interventions in reducing clinical features of depression, anxiety, stress and psychological components of pain alongside increases in well-being (Bohlmeijer et al. 2010; Chiesa and Serretti 2009; Chiesa and Serretti 2011; Grossman et al. 2004; Ledesma and Kumano 2009; Marchand 2012; Piet and Hougaard 2011). The two most popular programmes are mindfulness-based stress reduction (MBSR; Kabat-Zinn 1982) and mindfulness-based cognitive therapy (MBCT; Segal et al. 2002). Meta-analyses show moderate within-group effect sizes on clinical features associated with common psychological disorders (ES ≈ .5; e.g. Chiesa and Serretti 2011; Fjorback et al. 2011; Khoury et al. 2013).

The quality of the training provided by mindfulness teachers is considered crucial to the overall success of a mindfulness training programme (Crane et al. 2010). Up until recently, the majority of mindfulness teachers were, as Crane et al. (2012) describe it, first- or second-generation teachers who were either taught by Jon Kabat-Zinn or his colleagues, or learning from those who had. This is changing now and increasing numbers of mindfulness trainers are teaching with limited experience. In order to provide some benchmark for programmes, the UK Network of Mindfulness-Based Teacher Trainers (2011) provide an outline of the core experience and competencies which are considered an essential basis for teaching mindfulness. This includes teachers having a sustained and consistent personal mindfulness practice. Given the growing popularity of mindfulness-based approaches along with the extensive commitment and experience required to teach it, there is currently a tension between limited supply and increasing demand. There are concerns that in the rush to meet demand, it is possible that the integrity and authenticity of the work may be diminished if delivered by inexperienced teachers (Crane et al. 2012).

There is unlikely to be one single way to address this issue; however, one approach might be the delivery of well-designed mindfulness training online, using web-based programmes, mobile apps and other technological platforms. Internet-based interventions are by no means new (e.g. Barak et al. 2008; Taylor and Luce 2003); some are even well-established. Computerised cognitive behavioural therapy (cCBT), for example, is supported in the UK for use with mild to moderate depression and anxiety by the National Institute for Health and Clinical Excellence (National Institute of Clinical Excellence 2006). During cCBT, patients login to a secure website at specific time points in order to access and read content online. Content is generally arranged in a series of eight lessons or modules and can take the form of a website or even a smart-phone application (Andersson and Titov, 2014). Overall, there is evidence supporting use of such interventions for major depression, panic disorder, social phobia or generalised anxiety disorder, with a moderate mean within-group effect size of .88. (Andrews et al. 2010). However, there are a number of issues with these interventions. Drop-out rates can be high (range 3–34 %) and, as a recent meta-analysis suggested, effects may not be sustained into follow-up (So et al. 2013). Additional therapist support appears to be more effective than those without (ES = 1 vs .2; Spek et al. 2007) and reduce attrition rates (So et al. 2013), so there is some loss of effectiveness when direct contact with a therapist is lost.

By removing face-to-face contact with an experienced mindfulness teacher, interventions have the potential to be more cost-effective, accessible, and flexible. This needs to be balanced against the potential reduction in quality and ultimately effectiveness. Extensive research is therefore needed before such interventions can be supported for use in a clinical environment. It is clear that these interventions could be popular with participants. Commercial mindfulness training from websites and mobile apps (e.g. Headspace and Buddify) along with books with audio files is increasingly popular, and there is a growing public interest in mindfulness training online and using smart phones. Most of these products do not have any facilitator involvement beyond their initial design and many are similar to the interventions included in this review. This may offer cost-effective interventions with a huge potential for scalability after initial design.

This review explores the current research into mindfulness training interventions delivered on technological platforms that do not have direct facilitator involvement. We take a first look at whether such programmes are beneficial for participants and lead to changes to clinical outcomes such as stress, anxiety and depression which are consistent with the broader literature on mindfulness interventions.

## Method

Three databases (Ovid Medline, PsychINFO and Embase) were searched for relevant keywords, titles, medical subject headings (MeSH) and abstracts. Each database was searched up to the 6 July 2014. Two search strings were developed: (1) ‘mindfulness’ and (2) ‘technology’. Inclusion criteria were as follows: (1) the study intervention must teach formal mindfulness techniques (for example sitting and moving meditation as compared to informal mindfulness exercises such as being mindful of day to day activities), (2) the mindfulness training must be taught via technology only, (3) the study must include clinical outcomes, (4) the intervention must not include direct facilitation, (5) the paper must be available in full, and (6) it must be written in English. The titles and abstracts of identified studies were screened by two screeners (JF and JB). Disagreements were discussed. Data was extracted from the remaining papers using an electronic data extraction sheet. Any missing or unclear data was marked as such.

**Fig. 1:**
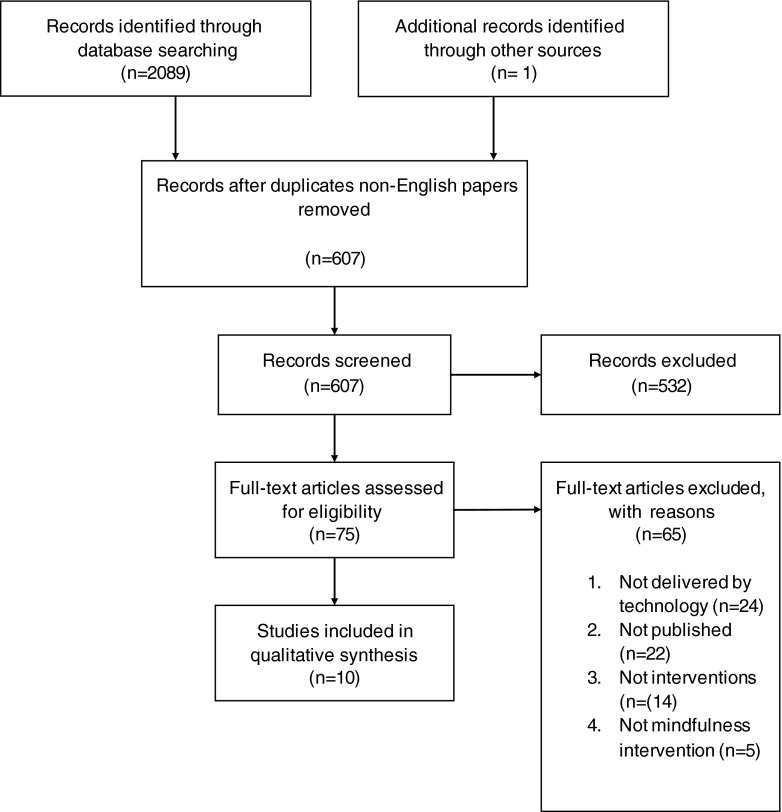
PRISMA flow diagram of mindfulness interventions delivered through technology without facilitator involvement (Moher et al. 2009)

A PRISMA flow diagram for this review demonstrates the process (see Fig. 1; Moher et al. 2009). Two thousand eighty-nine papers were initially identified after duplicates and non-English papers were removed. One thousand four hundred eighty-two papers were excluded after a title search, clearly on a different subject. A further 532 papers were excluded following abstract reading, clearly on a different subject. Sixty-five papers were then excluded after a full text review. Of these, 24 were not technology-delivered, 22 were not published research, 14 were not interventions and four were not mindfulness interventions. Nine studies remained. One paper that was in press was subsequently added (Dimidjian et al. 2014). This led to a total of 10 studies being included.

## Results

### Study Characteristics

Table 1 includes a summary of study characteristics. Nine of 10 studies were quantitative (Glück and Maercker 2011; Krusche et al. 2012; Altschuler et al. 2011; Krusche et al. 2013; Davis and Zautra 2013; Cavanagh et al. 2013; Boettcher et al. 2014; Dimidjian et al. 2014; Reid 2013). This included five RCTs (Glück and Maercker 2011; Davis and Zautra 2013; Cavanagh et al. 2013; Dimidjian et al. 2014; Boettcher et al. 2014) and four feasibility evaluations without controls (Altschuler et al. 2011; Krusche et al. 2012; Krusche et al. 2013; Reid 2013). No quantitative studies used the same sample although the qualitative study (Boggs et al. 2014) explored participant views following an intervention used in another quantitative study (Dimidjian et al. 2014).

**Table 1:** Summary of participant and intervention characteristics

Study	Mode of delivery	Control	Population	Effects measured	Intervention group characteristics		*N* (at start)	Course timing			Materials	Techniques	Reminders	Attrition (%)
					Mean age (SD/range)	Sex (F%)		Course duration	Total time commitment (excluding home practice)	Home practice (Instruction: *Measured*)				
Glück and Maercker (2011)	Web-based	Wait-list	Students	Depression, mindfulness, stress	33.7 (12.7)	71.4	49	13 days	4 h 20 min (13 × 20 min)	–	Written text, audio files, flash animation exercise	Awareness of bodily sensations, attention of breath, acceptance of emotions	Emails (before, halfway and after second module)	10.2
Krusche et al. (2012)	Web-based	-	Students	Stress	48 (11.25)	74	100	4–8 weeks (participant chooses pace)	Unclear	30-min formal + variable informal exercise per week: 33 % ‘every/most days’; 55 % ‘sometimes’; 12 % ‘rarely’	Audio, instructional videos, e-learning	Body scan, mindful movement, informal exercises	Emails	-
Altschuler et al. (2011)	Audio CD’s	-	Cancer patients	Anxiety, depression	56 (31.70)	91	23	12 weeks	20 h	5 days/week, 20 min each: *Av. of* 39 (4-60) *listens to audio CD*	Audio CDs, study diary	Awareness of breathing, body scan, loving kindness	Phone calls (3x a month)	48
Krusche et al. (2013)	Web-based	-	Students	Anxiety, depression, stress	47.7 (11.98)	78	273	4-8 weeks (participant chooses pace)	Unclear	1 30-min formal, 1 informal exercise per week:; 41 % ‘every/most days’; 52 % ‘sometimes’; 7 % ‘rarely’	Audio, instructional videos, e-learning	Body scan, mindful movement, informal exercises	Emails	-
Davis and Zautra. (2013)	Web-based	Health training	Patients with fibromyalgia	Depression, stress	46.14 (22.81)	98	79	6 weeks	3 h (12 × 15 min)	–	Visual presentation of text, animation and audio	Awareness and acceptance of emotions	-	15
Cavanagh et al. (2013)	Web-based	Wait-list	University students	Mindfulness, stress	25.28 (6.85)	90.7	104	2 weeks	Unclear	10 mins daily: *61* % >*1*/*day*; *87* % >1/*week*; *4* % *none*	Mindfulness education, audio, daily journal	Mindfulness of body, breath, thoughts and feelings	Emails (every 3 days)	52.3
Dimidjian et al. (2014) ^a^	Web-based	TAU	Individuals with recurrent depression	Anxiety depression, mindfulness	47.40 (11.43)	73	200	8 weeks	Unclear	Min 6 days practice per week: *Av. 11.25 h practising*	Video of interactions between instructors and participants, audio files	Sitting meditation, body scan, yoga, informal practices, 3-min breathing space	–	24
Boettcher et al. (2014)	Web-based	Discussion forum	General public with features of anxiety or depression	Anxiety, depression	37 (8.9)	75.6	91	8 weeks	16 h	Two times a day for 6 days per week; *Not measured*	Introductory video, audio files, psychoeducation	Sitting meditation, mindful movement, body scan, breathing anchor exercises	–	7.7
Reid (2013)	Web-based	-	Occupational therapy students	Mindfulness	-	-	15	8 weeks	Unclear	-	Audio files, informational readings, private journal	Sitting meditation, body scan, 3-min breathing space, mindful movement, loving-kindness, qigong	–	–
Boggs et al. (2014) ^a^	Web-based	-	Individuals with recurrent depression	-	46.89 (12.38)	71.1	-	8 weeks	Unclear	Minimum of 6 days practice per week: *Not measured*	Video of interactions between instructors and participants, audio files	Sitting meditation, body scan, yoga, informal practices, 3-min breathing space	–	24

Home practice is understood to mean practice of learnt meditations

*TAU* treatment as usual

^a^These studies used the same intervention

Participant numbers ranged from 15 (Reid 2013) to 273 (Krusche et al. 2013). Exclusion criteria were used in three studies: these included high levels of psychiatric disorder (Glück and Maercker 2011), symptoms consistent with current substance misuse (Dimidjian et al. 2014) and a history of more than five episodes of depression (Davis and Zautra 2013). Inclusion criteria were used in three studies: these included meeting diagnostic criteria for one of a number of psychiatric disorders (Boettcher et al. 2014) and a history of at least one major depressive episode (Dimidjian et al. 2014; Boggs et al. 2014).

The participants were all over 18 years of age; mean ages varied from 25 to 56 years. A gender bias was evident as female participants made up between 71 and 98 % of participants; this is similar to face-to-face interventions (e.g. Fjorback et al. 2011). The majority of studies used students or the general populations although more specific populations were studied which included patients with cancer (Altschuler et al. 2011), fibromyalgia (Davis and Zautra 2013) and individuals with recurrent depression (Dimidjian et al. 2014; Boggs et al. 2014).

### Intervention Characteristics

Table 1 includes a summary of intervention characteristics. Nine of the 10 programmes were primarily web-based, often with audio CDs with recordings of meditations). Altschuler et al. (2011) however used audio CDs only. The duration of the interventions ranged from 13 days (Glück and Maercker 2011) to 12 weeks (Altschuler et al. 2011). The total time commitment asked from participants was not always clear but where measured varied from three to 20 h. What practice of outside the main sessions was not clear in most studies. All interventions introduced participants to some form of mindfulness practice including body scans, mindfulness of breathing, mindful movement and loving kindness; some were much more extensive than others though. Reminders to practice were used in five studies using phone calls (Altschuler et al. 2011) and emails (Glück and Maercker 2011; Krusche et al. 2012; Krusche et al. 2013; Cavanagh et al. 2013).

Glück and Maercker (2011) used a web-based intervention aimed at reducing depression and stress along with increasing mindfulness in students. Over 13 days, participants were asked to take a 20-min module every day except one, totalling 4 h and 20 min. No mention of additional home practice was noted. Participant engagement was not measured. Written text, audio files and a flash animation exercise were used to teach and develop awareness of bodily sensations, mindfulness of breath and ‘acceptance of emotions’. Attrition was 10.2 %.

Krusche et al. (2012, 2013) used a web-based intervention aimed at reducing stress in students. The course ran for 4 to 8 weeks, the pace directed by the participant. The total time commitment was unclear. Home practice of one formal exercise lasting 30 min and one informal practice was asked for per week and participant engagement was measured; 12 and 7 % of participants ‘rarely’ practising in the 2012 and 2013 papers, respectively, all others doing exercises ‘sometimes’ (55/52 %) or every/most days (33/41 %). A modular format used instructional videos led by experienced mindfulness instructors and e-learning modules to teach a body scan, a sitting (it was unclear if this was akin to mindfulness of breath) and mindful movement meditations. Attrition rates were not stated and all statistical analysis was conducted with early completers so it could not be calculated post hoc.

Altschuler et al. (2011) used audio CDs to deliver an intervention aimed at reducing anxiety and depression in cancer patients receiving chemotherapy. The course ran over 12 weeks and the time commitment was 5 days a week for 20 min a day, totalling 20 h. Participants averaged 39 listens to the audio tracks, 13 h. The course consisted of two CDs recorded by a mindfulness instructor who had been successfully treated for cancer in the past: one CD was for patients to meditate during chemotherapy and the other was to be listened to at home. The tracks included awareness of breathing, a body scan and a loving kindness meditation. A reflective diary was used to record thoughts and engagement. Attrition rates were high at 48 %. Notably, this study did include one introduction session conducted in person. It was included in the review, as the rest of the study did not include contact with a group or facilitator.

Davis and Zautra (2013) used a web-based mindful socioemotional regulation intervention (MSER) aimed at reducing depression and stress in chronic pain patients. The course ran over 6 weeks and included 12 modules which lasted 15 min each, totalling 3 hours. The course consisted of written text, animations and audio to teach awareness and acceptance of emotions. Details of specific techniques or home practice were not reported. The attrition rate was 15 %.

Cavanagh et al. (2013) used a web-based intervention aimed at reducing stress and increasing mindfulness in university students. The course ran over 2 weeks and consisted of 10-min daily sessions, totalling 2 h 20 min. Audio files recorded by clinical psychologists who had training and experience with face-to-face mindfulness courses taught participants mindfulness of breathing, body and thoughts and feeling separately. The intervention also included a frequently asked questions page, daily journal and study information. Home practice was not noted. Attrition was 52.3 %.

Both Dimidjian et al. (2014) and Boggs et al. (2014) used the same intervention. Mindful mood balance (MMB), adapted from MBCT, aimed to reduce anxiety and depression whilst increasing mindfulness in individuals with recurrent depression. The intervention ran over 8 weeks and asked participants to practice for a minimum of 6 days a week for 20 min a day, totalling 16 h. Participants reported an average of 11.25 h out of a total of 16 possible hours. The course used videos of interactions between instructors and participants and audio files to teach sitting meditations, body scans, yoga, 3-min breathing space exercises and informal practice. Attrition was 24 %.

Boettcher et al. (2014) used a web-based intervention to reduce anxiety, depression and insomnia in members of the public with features of anxiety or depression. The intervention ran over 8 weeks and asked participants to practice for 10 min twice a day 6 days of the week. The course was organised into eight modules with brief, instructive audio files teaching mindfulness of breathing, mindful movement and body scans. At the start of training, participants were presented with a 20-min educational video on mindfulness and anxiety. Attrition was 7.7 %.

Reid (2013) study used a web-based intervention to increase mindfulness in occupational therapy students. The course ran for 8 weeks and appeared similar to MBSR in content. The level of participation and engagement is unclear. The study stated that it expected students to spend a ‘few hours a week’ on the intervention but further details or measurement was not noted. A private journal was used to enable students to document their reflections and concerns regarding their experiences in the previous week. Attrition rate was not noted.

### Main Outcomes

#### Stress

Encouragingly three of the five studies that included well-validated stress measures reported moderate or large effects (see Table 2), *d* = .62 (Cavanagh et al. 2013), 1.20 (Krusche et al. 2012) and 1.57 (Krusche et al. 2013). In both of their studies, Krusche et al. group included only self-selected quick completers of the intervention in analysis; therefore, these results are likely to suggest the upper limits of effects. Two other studies found small or non-significant effects (Davis and Zautra 2013; Glück and Maercker 2011). There studies had notable issues with intervention and study design (e.g. small participant numbers, less theoretical basis to intervention and unvalidated measures). Whether changes have a clinical correlates is difficult to determine as they do not translate easily. However, one study did look at the real significance of change (RCI) and found that, based on the perceived stress questionnaire (PSQ), the intervention group was nine times more likely to improve, based on a deviation from the mean of 1.96, compared to the waitlist group (Glück and Maercker 2011). Comparisons of the interventions to alternatives were made in three studies. The largest and highest quality study found a moderate difference between the intervention and a waitlist, *d* = .42 and *p* < .005 (Cavanagh et al. 2013). Other studies, of poorer quality, found very small or non-significant differences compared to health training and waitlist (Glück and Maercker 2011; Davis and Zautra 2013).

**Table 2:** Intervention effects on stress, depression, anxiety and mindfulness measures

Study	Dose: practice level (*Reported*)	Control group	Measure		T1 (baseline)	T2 (post)	T3 and 4 (FU)	Effect sizes	
								BG	WG
Glück and Maercker (2011)	20 min/day (*not measured*)	Wait list	Stress	PSQ	40.06 SD = 16.38	34.36 SD = 15.06	7MFI 27.89 SD = 11.18	I < C(T2): ns	T1 > T2: ns
			Depression	SEK-27	2.70 SD = 0.48	2.81 SD = 0.54	7MFU 3.00 SD = 0.59	ns	Ns
				PANASneg	1.48 SD = 0.93	0.96 SD = 0.70	7MFU 0.97 SD = 0.48	ns	*d* = .43** Maintenance unclear
				PANASpos	2.53 SD = 0.59	2.54 SD = 0.79	7MFU 2.93 SD = 0.61	ns	T1 = T2 T2 < T3: d = 0.43*
			Mindfulness	FMI	37.04 SD = 5.37	38.77 SD = 6.46	41.16 SD = 6.05	I = C(T2): ns	T1 < T2: *d* = 0.32** T2 < T3: *t*(27) = 1.980*
Krusche et al. (2012)	30 min one time a week (33 % ‘every/most days’; 55 % ‘sometimes’; 12 % ‘rarely’)	–	Stress	PSS	23.04 SD = 6.85	15.06 SD = 6.42	13.45 SD = 6.99	–	T1 > T2: *d* = 1.20** T2 > T3: *d* = 0.24**
Altschuler et al. (2011)	20 min 5 days a week (*average 16*)	–	Depression	HADS-D	6.7 normal	4.5 normal	–	–	–
			Anxiety	HADS-A	11.5 CI 9.9–13.0	6.7 CI 4.6–8.8	–	–	–
Krusche et al. (2013)	30 min oe time a week (33 % ‘every/most days’; 55 % ‘sometimes’; 12 % ‘rarely’)	–	Stress	PSS	23.73 SD = 49.95	14.44 SD = 45.86	1MFU: 13.45 SD = 6.99	–	T1 > T2 = T3: *d* = 1.57**
			Depression	PHQ-9	10.06 SD = 6.40 moderate	5.04 SD = 3.84 mild	1MFU: 4.30 SD = 3.97 mild	–	T1 > T2: *d* = .95** Further improvement at 1MFU, *d* = .19**
			Anxiety	GAD-7	10.98 SD = 6.42	5.45 SD = 3.77	4.60 SD = 3.72	–	T1 > 2: *d* = 1.22** T2 > 3: *d* = .24*
Davis and Zautra. (2013)	15 min two times a week	Health training	Stress	Family-related stress	1.99 SD = 1.01	–	–	I < C(T2): *d* = .006?	–
				Stress Coping efficacy	3.12 SD = .96	–	–	I < C(T2): *d* = .014?	–
			Depression	Positive and negative effect (Likert)	Positive: 2.29 SD = 1.05 Negative 2.20 SD = 0.92	–	–	Positive I > C (T2): *d* = 0.06* Negative I > C (T2): *ns*	Positive T1 > 2: unclear *ns* Negative T1 > 2: unclear**
Cavanagh et al. (2013)	10 min everyday (*61* % >*1*/*day*; *87* % >*1*/*week*; *4* % *none*)	Wait list	Stress	PSS	21.70 SD = 7.87	18.96 SD = 6.75	–	I < C(T2): *d* = .37**	T1 < T2 *d* = 0.42**
			Mindfulness	FFMQ	117.76 SD = 21.48	123.66 SD = 20.16	–	I > C(T2): *d* = 0.27*	T1 < T2 *d* = 0.42**
Dimidjian et al. (2014)	20 min 6 days a week (Average 1 h 20 min a week)	TAU	Depression	PHQ-9	4.21 SD = 3.03 mild/minimal	2.89 SD = 2.69 minimal	10WFU 3.40 SD = 3.01 minimal 6MFU 3.04 SD = 2.53 minimal	I < C(T2): *d* = 0.78**	T1 > T2: *r* = 0.27** Maintained at 10W and 6MFU
			Anxiety	RSQ	45.12 SD = 13.67	40.45 SD = 15.11	–	–	T1 < T2: *r* = -23**
			Mindfulness	FMI	126.52 SD = 18.42	132.35 SD = 18.42	–	–	T1 < T2: *r* = 0.52*
Boettcher et al. (2014)	20 min 6 days a week (not measured)	Discussion forum	Depression	BDI-II	16.4 SD = 7.0 mild	6.5 SD = 4.8 minimal	6MFU 9.6 SD = 8.2 minimal	I < C(T2): *d* = 0.84*	T1 > T2: *d* = 1.58** Post < 6MFU*
			Anxiety	BAI	24.4 SD = 8.6	11.8 SD = 7.8	6MFU: 12.4 SD = 6.7	T2: *d* = .99**	T1 > 2: *d* = 1.33** T1 > T2: *d* = 1.44**
Reid (2013)	Not reported	–	Mindfulness	MAAS	51.73 SD = 8.22	61.72 SD = 5.44	–	–	T1 < T2: *t* = −4.82**

*C* control, *I* intervention, *FU* follow-up

*p < .05, **p < 0.01

#### Depression

Three of the six studies including well-validated depression measures reported statistically significant reductions with clear clinical correlates (Dimidjian et al. 2014; Boettcher et al. 2014; Krusche et al. 2013). Scores indicative of ‘mild’ depression were reduced to ‘minimal’ (Dimidjian et al. 2014; Boettcher et al. 2014) and ‘moderate’ to ‘mild’ (Krusche et al. 2013). Other studies were less indicative of real clinical change, but some did find reductions in depression scores. One study employed an unvalidated depression measure (Davis and Zautra 2013); another used a measure without guidelines in English (Glück and Maercker 2011). One study used a validated measure but did not calculate significance (Altschuler et al. 2011). One study tested for real clinical indicators of change (discussed earlier) and found no significant change on PANAS or SEK-27 measures that relate to depression (Glück and Maercker 2011). With promising results, four studies compared the intervention to alternatives. Two studies reported large and significant reductions in post-intervention scores compared to waitlists, *d* = .78 (Dimidjian et al. 2014) and *d* = .84 (Boettcher et al. 2014). Two studies found very small or non-significant effects of the mindfulness intervention compared to health training (Davis and Zautra 2013) and waitlist (Glück and Maercker 2011).

#### Anxiety

The two studies which tested for significance on anxiety measures found significant changes. These were large and have clear clinical correlates after interventions. Scores indicative of ‘moderate/severe’ anxiety were reduced to ‘mild’ (Boettcher et al. 2014) and ‘moderate’ to ‘mild’ (Krusche et al. 2012). Another study found an average reduction from ‘mild/moderate’ to ‘normal’ but did not test significance (Altschuler et al. 2011). A significant reduction in rumination was noted in one study although size or clinical correlation was omitted (Dimidjian et al. 2014). One study compared an intervention to an alternative (a discussion forum) and found a large difference (*d* = .99, *p* < 0.01; Boettcher et al. 2014)

#### Mindfulness

All four studies including a mindfulness measure found significant increases. Changes ranged statistically from small to moderate in size on the FFMQ (Cavanagh et al. 2013) and the FMI (Glück and Maercker 2011; Dimidjian et al. 2014). One study found a significant increase the MAAS that was not sized (Reid 2013). Note that some studies investigated other effects but they have not been included in this review (e.g. insomnia in Boettcher et al. 2014).

### Longevity of Effects

Two of the studies explored continued practice of mindfulness after the intervention ended. One study reported that at 3-month follow-up, 50 % of participants continued to practice when they felt stressed and 25 % maintained regular practice (Glück and Maercker 2011). Another study found that 83 % of participants reported that they intended to continue practice on a regular basis (Cavanagh et al. 2013). In many studies, effects either lasted into follow-up or even further increased. Stress reduction was maintained or further reduced in all three studies which investigated this: maintained at 1 and 3 months (Krusche et al. 2012; Glück and Maercker 2011) and further reduced at 1 month (Krusche et al. 2013). Continued effects on depression measures were present in three of four studies which investigated this. Two studies had maintained effects at 6 months (Boettcher et al. 2014; Dimidjian et al. 2014) and one a further small significant decrease at 1 month (Krusche et al. 2013). Conversely, one study found no effect present at 6 months (Glück and Maercker 2011). Effects on anxiety appear to last until at least a month after intervention, although only two studies conducted follow-up analyses. Reductions were maintained at 6 months in one study (Boettcher et al. 2014) and even further reduced at one month in another (Krusche et al. 2013). The only study which included a follow-up measure for mindfulness found a marginally significant further increase in scores at 7 months (Glück and Maercker 2011).

### Attrition, Participation and Engagement

It is clear that participants did not always fully commit to the programmes, either engaging less than instructed or even dropping out. The reasons for this are unclear. Levels of ‘home practice” (that outside of first teaching of a technique) varied from none (noted) up to 20 min a day. The largest effects were found in the Krusche et al. studies which encouraged 30 min once a week of formal practice plus an informal exercise (many participants achieving more than this), but as mentioned earlier due to study design flaws, these are likely to represent the maximal effects. As one would expect, studies which conducted statistical analyses with only participants who completed the intervention with a high degree of participation and engagement (Krusche et al. 2012; Krusche et al. 2013; Dimidjian et al. 2014) found greater effects on outcomes than those that did not. Longer course completion times, suggestive of less participation and engagement, correlated with weaker effects on outcome measures in one study (Krusche et al. 2013). Specifically, informal practice (i.e. integration of mindfulness into daily activities), but not formal (e.g. sitting meditations), was correlated with those outcomes. Reported attrition rates ranged from from 52.3 % (Cavanagh et al. 2013) to 7.7 % (Boettcher et al. 2014). Both of these courses were similar and that was a reflection of the lack of clear differences between courses that were better at retaining participants. Interestingly, Glück and Maercker (2011) found that participants with higher levels of initial distress were less likely to drop out.

### Participant Feedback

Three studies measured the subjective benefit of participation with broadly positive findings. In one study, training was ‘beneficial’ for 73.5 % of participants after the interventions and 66.6 % at 3-month follow-up agreed. A total of 70.3 % felt the intervention ‘helped inner balance’ and 77.2 % would ‘recommend’ the web-based training (Glück and Maercker 2011). Another study reported that 87 % of participants thought the programme was ‘at least some benefit’ although 13 % thought it was ‘no benefit at all’ (Cavanagh et al. 2013). Altschuler et al. (2011) reported that participants were ‘very positive’ about participation and perceived that the intervention helped them ‘cope better with diagnosis and treatment’, although no further details were given.

Only one study gives us some insight into participant experience of an intervention. The qualitative study (Boggs et al. 2014) included in this review can highlight what may be strengths of existing interventions along with potential improvements. The Boggs et al. (2014) study used MBCT as a basis for its online modular format and identified four themes in participant responses: evidence of concept comprehension, home practice, MBCT web content, and MBCT web-based group process. All of the participants seemed to grasp what mindfulness was and found an overall positive impact of the intervention. Many gave practical examples of putting learnt concepts into practice. Participants noticed an increased sensitivity to personal warning signs of imminent depressive relapse and successfully implemented mindfulness practices to counteract these symptoms. Time constraints and lack of motivation were reported to prevent completion of the recommended home practice at times. Many aspects of the intervention were positively received. The modular e-learning format of the programme was clear and text outlined key concepts in an effective manner. Participants spoke very highly of the videos of group leaders speaking. This was perceived as a good alternative to written text for some individuals. Audio recordings of guided meditations were perceived as helpful, and most stated they would use them in the future. Some aspects of the intervention were not perceived positively by some participants: particular mediations, especially the body scan, practice requirements (30 min from the start) and an absence of an instructor or group. Some alternative approaches were suggested such as starting on 15 min and gradually increasing practice requirements. One programme is not likely to fit all participants. For example, in opposition to others, a small number of participants stated they were not ‘group learners’. The group videos seemed to be an adequate alternative for many of the participants, who felt that they got to know the group in the videos.

## Discussion

Although research is in its infancy, this review lends some initial support to mindfulness interventions using technological platforms to deliver mindfulness training without direct facilitator input. Reduction in clinical features of anxiety, depression and stress was found in a number of studies. Anxiety and depression effects were comparable in size to NICE recommended cCBT (Andrews et al. 2010) along with face-to-face mindfulness interventions (within group ES ≈ .5; e.g. Chiesa and Serretti 2011; Fjorback et al. 2011; Khoury et al. 2013). Effects appear to last into follow-up, even increased further in one study at 1-month post-intervention. Despite encouraging findings, firm conclusions about effectiveness cannot be made yet.

Criticisms of cCBT that dropout rates can be high and effects may not be sustained (So et al. 2013) support the need for research into other interventions like those reviewed which may produce longer lasting effects and lower attrition rates. The mindfulness interventions included had a wide range in dropout rates (7.7-52.3 %) so cannot currently be favourably compared to cCBT in terms of attrition. However, there is initial support for the longevity of stress, anxiety and depression reduction effects. Not only were continued effects seen in the majority of studies which measured this but also one study even found further effects after 1 month. Notably, this study was subject to multiple biases in its design, using fast completers only in analysis. Longevity of effects may be due to the nature of mindfulness interventions which teach the use of continued formal and informal practice n a way that CBT does not.

Participation, engagement and attrition are all likely to play an important role in outcomes and mediate effects. A dose-response relationship may even exist although the evidence so far is not strong enough to support this. One finding by Krusche et al. (2012) that longer course completion times correlated with weaker effects on outcome measures supports this. Specifically, the contribution of informal but not formal practice lies in opposition to a previous study by Carmody and Baer (2008) who found no significant relationship between informal practice and outcomes in their review of face-to-face interventions.

Participant and intervention characteristics are likely to both contribute to effects. Whether participant characteristics led to greater effects in certain interventions is largely unclear although one study found that participants with higher levels of initial distress had lower attrition rates (Glück and Maercker 2011). Participants that urgently require help may be more likely to actively engage with practice and gain greater effects. Unfortunately, none of the studies investigated why people left studies. Course length and time commitment, encouraged daily practice, meditations taught (e.g. body scans, mindfulness of breathing and mindful movement) and types of resources (e.g. text, audio, videos and workbooks) all varied between studies and there is no clear link between these and outcomes. Despite the lack of clear support for one particular model though, shared characteristics, especially between the more successful interventions, do suggest a template on which future interventions should be based. The qualitative study found that participants liked many of the elements shared between interventions, particularly the web-based, modular e-learning format that used different types of content such as text, audio and videos. Animations were less popular.

Whilst these programmes do not include direct facilitator contact with participants, it is important to recognise the role of those who designed the programme and prepared the course materials. Experience of those who designed the course was not mentioned in most papers but this is also likely to play an important role. Given that quality of the training is thought to be crucial to the effectiveness of face-to-face mindfulness training (Crane et al. 2010), it stands to reason that those involved in an online programme are just as, if not more, important as the trainer in a face-to-face mindfulness intervention. Programme developers are doing two things, developing a targeted teaching programme for a given population and then deciding the best way to communicate and deliver that using technology. Recently, the UK Network of Mindfulness-Based Teacher Trainers (2011) developed good practice guidelines for those delivering mindfulness training face-to-face. It seems important that the conversation now extends to the development and delivery of mindfulness programmes online, learning programmes, and fully online training programmes.

One concern about technology delivered mindfulness programmes such as those reviewed is that the integrity and authenticity of the teachings will necessarily be compromised given the lack of face-to-face contact with a facilitator. This review found evidence that interventions can teach mindfulness and maintain faithfulness to accepted models. The qualitative study included (Boggs et al. 2014) went some way to show that it is at least possible for such interventions to teach the core elements of face-to-face courses. For example, participants here stated that they were better at coming out of ‘autopilot’ and ‘slowing down’ along with re-framing negative thoughts and identifying triggers for anxiety and depression. Quantitative support comes from all four studies that assessed mindfulness which reported increases in mindfulness. There at the very least exists a correlation between clinical effects and mindfulness levels.

It is vital that we learn how to make interventions more effective, and one way to do this may be by encouraging more practice. Practice times were short across the studies and no interventions encouraged or achieved levels of practice seen on a face-to-face MBSR/CT course (40 min a day). However, participants in the qualitative study have noted that the amount of time encouraged (30 min) is too much, especially at the start of the intervention (Boggs et al. 2014). This may explain some of the attrition. Notably, many studies found comparable rates to those found with face-to-face interventions (16.5 %; Khoury et al. 2013). Shortening practice times may achieve lower attrition rates and more participation and engagement, but they may also reduce the effectiveness of the intervention. One way forward might be to encourage multiple shorter sessions throughout the day building on the 3-min breathing space included in many programmes already. Another option may be to gradually increase time commitment as suggested by participants in Boggs et al. (2014).

Moving forward, research is likely to follow two distinct paths. The first of which is to build on the initial support for the effectiveness of technology-delivered mindfulness interventions without direct facilitator input. This should also include an understanding of mechanisms behind effective interventions. A standardised intervention format would allow for robust, large-scale quantitative evaluations and adaptations including novel approaches. Previous interventions and quantitative study suggest elements which should be used in the future including a modular course structure, use of different materials within the same course (e.g. text, audio, videos and printouts) and an e-learning environment. The importance of many key elements such as course length, practice time, materials used, techniques taught and course designer experience are still not well understood, and comparisons between otherwise standardised interventions would elucidate the impact of these elements. This approach also allows specific interventions focus on particular issues (e.g. addiction, depression and eating disorders). An approach incorporating standardisation and specific focus of interventions is similar to that seen in the face-to-face MBSR/MBCT community (e.g. Kristeller and Wolever 2011; Sibinga et al. 2011; Salmoirago-Blotcher et al. 2012; Reid 2013). High-quality supporting research is needed.

This review recommends a number of components to a standardised intervention model: courses should last at least 4 weeks; 30 min of practice for 6 days a week should be encouraged although variations on this should be explored; the use of a wide range of materials (e.g. audio, video and text), development find experienced mindfulness practitioners should develop courses; core mindfulness exercises should be taught (i.e. mindfulness of breathing and movement, body scans). This review also recommends a number of components to future research models: conducting intention-to-treat analyses, comparing interventions against appropriate control groups (e.g. cCBT or face-to-face mindfulness interventions), measuring mindfulness as a mediator, including follow-up measures up to at least 6 months, measuring individual practice levels and drop-out rates and including exploration of participants’ experiences, especially their understanding of mindfulness and why some participants drop out or do not fully engage. A limited number of well developed programmes must be comprehensively evaluated before widespread use.

The second research focus should be experimentation with novel approaches including integration of interventions with face-to-face programmes and varying degrees of instructor-led or group work through technology. The use of mobile technology may offer an innovative way to encourage practice. Many existing interventions already included reminder emails. Building on this, mobile technology could also be used to monitor and encourage home practice. Like web-based delivery, it is low cost and flexible. Use of new technologies such as mobile apps and even integration of technology into established programmes could create more effective interventions. Although some of the advantages of a self-contained intervention, such as low cost, scalability and accessibility, may be lost, technology may be a useful addition to existing face-to-face interventions within a blended learning environment. This would satisfy the need that some participants, but notably not all, have for facilitator and group interactions. Another approach may be to have groups that meet to share in online delivery of interventions designed by experienced teachers, perhaps even with live video links.

### Limitations

Caution should be taken before any conclusions on effectiveness of these interventions are made. Some studies had small or insignificant measured effects and the studies with larger effects had a number of methodological weaknesses. Krusche et al. (2012, 2013) found the largest effects but shared methodological flaws with other studies that prevent more general support of these interventions being made (other studies referenced in brackets). Krusche et al. used completers only in analysis (Boggs et al. 2014) and lacked follow-up (Cavanagh et al. 2013; Reid 2013; Davis and Zautra 2013) and perhaps most importantly, they did not have a control group (Altschuler et al. 2011; Reid 2013). No studies used a similar intervention (e.g. cCBT, counselling or face-to-face mindfulness interventions) as a control which is another major limitation of research so far. The high proportion of women along with clinical or student populations in study groups may also limit generalisability. This review does not include a meta-analysis so overall effects, including those of potential mediating variables, have not been calculated.
